# Redox Modulation at Work: Natural Phytoprotective Polysulfanes From *Alliums* Based on Redox-Active Sulfur

**DOI:** 10.1007/s40495-018-0153-2

**Published:** 2018-09-21

**Authors:** Awais Anwar, Emma Gould, Ryan Tinson, Javaid Iqbal, Chris Hamilton

**Affiliations:** 1 Ecospray Limited, Grange Farm, Hilborough, Thetford, Norfolk, IP26 5BT UK; 2 School of Pharmacy, University of East Anglia, Norwich Research Park, Norwich, NR4 7TJ UK; 3 Department of Plant Protection, College of Food and Agriculture Sciences, King Saud University, Riyadh, Saudi Arabia

**Keywords:** Garlic, *Allium sativum*, Polysulfanes, Reactive sulfur species, Glutathione, Redox modulators, Chemo-prevention

## Abstract

**Purpose of review:**

This article provides a brief overview of natural phytoprotective products of *allium* with a special focus on the therapeutic potential of diallyl polysulfanes from garlic, their molecular targets and their fate in the living organisms. A comprehensive overview of antimicrobial and anticancer properties of published literature is presented for the reader to understand the effective concentrations of polysulfanes and their sensitivity towards different human pathogenic microbes, fungi, and cancer cell lines.

**Recent findings:**

The article finds polysulfanes potentials as new generation novel antibiotics and chemo preventive agent. The effective dose rates of polysulfanes for antimicrobial properties are in the range of 0.5–40 mg/L and for anticancer 20–100 μM. The molecular targets for these redox modulators are mainly cellular thiols as well as inhibition and/or activation of certain cellular proteins in cancer cell lines.

**Summary:**

Antimicrobial and anticancer activities of polysulfanes published in the literature indicate that with further development, they could be promising candidates for cancer prevention due to their selectivity towards abnormal cells.

## Introduction

Sulfur plays a major role in biology and is found in numerous peptides, proteins, and low molecular weight metabolites. Among the sulfur compounds found in plants, bacteria, fungi, and animals are many agents with unique chemical and biochemical properties linked to redox processes, metal binding, and catalytic reactions to name a few.

Nature provides a range of sulfur redox modulators from plants, fungi, bacteria, and animals that have been investigated to determine their therapeutic potential. Of which the genus *allium* presents a range of sulfur-based natural products with many benefits to human health from antimicrobial to anticancer effects. These natural products are particularly prominent in garlic (*Allium sativum*) and onion (*Allium cepa*) mainly consisting of thiosulfinates and polysulfanes. The biological activity of such compounds is often associated with a broad spectrum of (bio)chemical properties. Their modes of action are often associated with redox activity, catalysis, metal binding, enzyme inhibition, and/or radical generation allowing these reactive sulfur species (RSS) to interact with oxidative stressors, to affect the function of redox-sensitive cysteine proteins, and to disrupt the integrity of DNA and cellular membranes. This has been discussed in various reviews previously [1, 2]. In some cases, the biological activity of sulfur-containing plant products depends on initial enzymatic activation, which allows thiosulfinates to be generated with high target selectivity. The antibiotic and anticancer activities of RSS make them interesting from a pharmacological perspective. Not surprisingly, research into the biochemical and pharmacological properties of these sulfur chemotypes is advancing rapidly especially as anticancer agents [3].

In order to understand how these sulfur compounds develop their biological activities, we need to consider the rather complicated chemistry of various sulfur chemotypes and their biochemical transformations. Sulfur redox networks provide a glimpse of sulfur-centered formation and transformation pathways in vivo [4]. Although such networks are continuously expanding, they serve as a snapshot of the sulfur redox chemistry known to date and illustrate the complexity of redox-active sulfur species. For full details on sulfur redox mechanisms and pathways, a previous review article by Jacob is worth reading to understand this chemistry [2].

### Redox Sulfanes of *Allium*

Sulfur metabolism in plants provides a treasure of reactive sulfur species (RSS) that includes several chemically unusual substances, such as thiosulfinates and polysulfanes from *Alliums*.

**Fig. 1:**
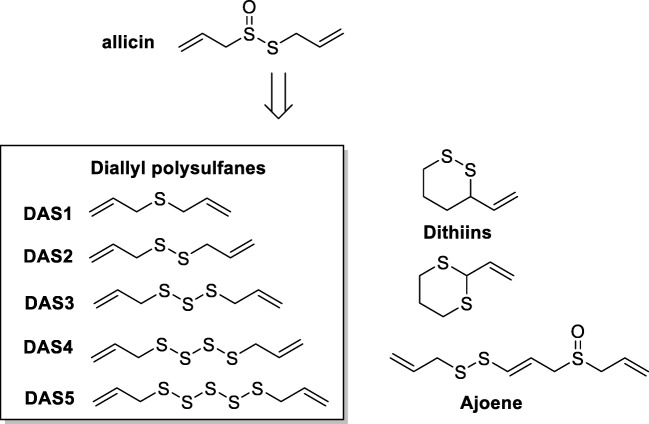
A selection of the redox-sulfur chemistry found in garlic. Garlic produces various other chemicals which are not part of this review. The polysulfanes produced by garlic on crushing vastly depend on methods of extraction and temperature [5]. In figure DAS1 (diallyl sulfane), DAS2 (diallyl disulfane), DAS3 (diallyl trisulfane), DAS4 (diallyl tetrasulfane), DAS5 (diallyl pentasulfane)

Polysulfanes are the most abundant organosulfur metabolites produced by garlic (Fig. 1) and are the result of an enzymatic reaction involving the non-protein amino acid alliin (*S*-allyl-l-cysteine sulfoxide), stored in large amounts in the cytosol of the plant cells (5–14 mg/g fresh dry weight, 1.4% of fresh weight) and an enzyme, allinase, present in the vacuole [6]. Upon crushing the enzyme substrate reaction produces and intermediate thiosulfinates, allicin which is not very stable at room temperature (half-life 3.1 h at 20 °C) [7].

Upon heating, allicin undergoes a cascade of further chemical rearrangements leading to other organosulfur molecules such as ajoene, dithiines, and predominantly diallyl polysulfanes (Fig. 1). The diallyl polysulfanes with DAS1-DAS4 constitute a major part of garlic extract and oils with others like methylated polysulfanes [8]. In this article, we will limit discussion to the diallyl polysulfanes mentioned in Fig. 1.

These diallyl polysulfanes (DAS1-DAS6) depicted in Fig. 1 exhibit distinct redox properties, which provide an interesting spectrum of biological activities in vivo, such as antibiotic, fungicidal, pesticidal, or anticancer activity. The last decade has provided an insight into the molecular basis for such activity and has achieved a better knowledge of the in vitro properties of diallyl polysulfanes. This has led to an improved understanding of their impact on intracellular redox signaling and control pathways in living cells.

The full impact of sulfur in living systems becomes apparent by considering the diversity of sulfur species and their reactions. The biological activity of RSS can be attributed to sulfur and its uniqueness to exist in various oxidation states that exist naturally and able to transform in in vivo environments to plethora of different chemotypes. The oxidation states and various chemotypes have been discussed in a detailed review previously [2]. The redox-active sulfur species are able to oxidize thiols to generate oxidative stressors (e.g., peroxides, hydroxyl radicals, hydropersulfides, persulfides) which in turn results in cocktail of RSS that can adopt various pathways in the cell.

### Medicinal/Pharmacological Properties of Sulfanes

#### Antimicrobial Activity

The key studies accredited allicin as the main contributor for the antimicrobial activity of garlic [9]. Allicin was found to inhibit bacterial growth as a vapor of lung pathogenic bacteria from the genera *Pseudomonas*, *Streptococcus*, and *Staphylococcus*, including multi-drug-resistant (MDR) strains, suggesting that it could be used to combat bacterial lung infections via direct inhalation; currently, there are no volatile antibiotics available to treat pulmonary infections [10]. Growth inhibition of *Escherichia coli* during allicin exposure coincides with a depletion of the glutathione pool and *S*-allylmercapto-modification of proteins, resulting in an overall decreased total sulfhydryl levels, which is accompanied by the induction of the oxidative and heat stress response. The mode of action of allicin is a combination of a decrease of glutathione levels, unfolding stress, and inactivation of crucial metabolic enzymes through *S*-allylmercapto-modification of cysteines [11]. It is suggested that allicin’s ability to permeabilize cell membranes may contribute to its antimicrobial activity independently of its activity as a thiol reagent [12].

Garlic oil and its major diallyl polysulfanes constituents, as well as garlic extract, and allicin possess significant activity against *H. pylori* [13]. DAS2 and DAS3 were found to be the most abundant components in garlic oil. Interestingly, DAS4 also inhibits *H. pylori*, an activity which might be relevant in the context of garlic consumption and stomach ulcers. The minimum inhibitory concentrations (MICs) and minimum bactericidal concentrations (MBCs) of DAS4 against the NCTC 11637, and 107018 B strains of *H. pylori* are between 3 and 6 μg/mL, i.e., even lower than the values for allicin (6–12 μg/mL). DAS1, DAS2, and DAS3 were less active, with MIC values of 2074–4148, 100–200, and 13–25 μg/mL, respectively [14].

There have been many studies using garlic to combat clinically important bacteria and fungi. Garlic extract was tested against three major antibiotic resistant pathogens: *C. albicans*, MRSA, and *P. aeruginosa*. Interestingly, a synergism effect was reported of garlic extract improving the effectiveness of the antibiotics [15]. A separate study on 30 clinical isolates of MRSA found that allicin, extracted from garlic, caused 88% of the strains tested to have MICs of 16 μg/mL and all strains were inhibited at 32 μg/mL [16]. Garlic extract was used in a study on the drug resistant pathogens *E. coli*, *P. aeruginosa*, *B. subtilis*, *S. aureus*, *K. pneumoniae*, *S. sonnei*, *S. epidermidis*, and *S. typhi.* All pathogens showed high susceptibility to garlic extract where the lowest MIC was 0.05 mg/mL [17]. Studies using synthetic allicin, which included multi-drug resistant (MDR) strains, showed that the growth of the majority of *Pseudomonas*, *Streptococcus*, and *Staphylococcus* isolates was completely inhibited by 64 μg/mL allicin. *S. pyogenes* (SNo 67467), *S. pneumoniae* (SNo 68668), and *S. aureus* (ATCC 43300) were completely inhibited by 32 μg/mL allicin and all *A. baumannii* isolates were completely inhibited by 16 μg/mL. However, *K. pneumoniae* isolates were slightly more resistant, with a MIC of 128 μg/mL. *P. aeruginosa* (DSM2659) showed high resistance to allicin (MIC 512 μg/mL) compared to *P. aeruginosa* (PAO1 SBUG8 and PAO25), MIC 64 μg/mL. MDR and non-MDR *S. pneumoniae* strains tested were equally susceptible to allicin and showed MICs from 32 to 64 μg/mL allicin and MBCs from 64 to 128 μg/mL allicin, respectively. This study shows that different strains have different susceptibilities to garlic and its constituents [10].

Antifungal studies found that garlic oil can penetrate the cell membrane of *C. albicans* as well as organelle membranes, such as mitochondria, which would result in destruction of the organelle and ultimately cell death. Due to their lipophilic nature, it is likely that many of the diallylpolysulfanes can pass through membranes of various organisms [18] and they have been shown to interact with membrane lipids to modify membrane fluidity [19].

Garlic oil has been shown to induce differential expression of important genes such as those involved in pathogenesis, oxidation-reduction, and cellular response to drugs and starvation [20]. Allicin and aged garlic extracts exhibit antimicrobial properties against the *Burkholderia cepacia* complex (Bcc), an intrinsically multi-resistant and life-threatening human pathogen showing the modification of cysteine residue which suggest allicin ability as a general electrophilic reagent targeting protein thiols [21, 22].

An important observation, which is demonstrated in Tables 1 and 2, is the different susceptibilities of pathogens to the different garlic constituents. DAS3 and DAS4 from garlic exhibit a wide spectrum of antimicrobial, antibacterial and antifungal activities For example, DAS3 and DAS4 both inhibit *S. aureus* (MIC 2.0 and 0.5 μg/mL, respectively), *S. aureus* (MRSA) (MIC 8.0 and 2.0 μg/mL, respectively), *C. albicans* (MIC 1.0 and 0.5 μg/mL, respectively), *C. krusei* (MIC 8.0 and 4.0 μg/mL, respectively), *C. glabrate* (MIC 4.0 and 2.0 μg/mL, respectively), *A. niger* (MIC 2.0and 1.0 μg/mL, respectively), *A. fumigatus* (MIC 8.0 and 4.0 μg/mL, respectively), and *A. flavus* (MIC 4.0 and 2.0 μg/mL, respectively). While the DAS3 is consistently less active, DAS4 possesses an antibiotic activity comparable to that of allicin. For example, against *H. pylori*, DAS4 has a MIC of 3–6 mg/L and allicin has a MIC of 6–12 mg/L [23].

**Table 1:** MIC values (mg/L) of polysulfanes against different human pathogenic bacteria. MIC values have been converted to mg/L where other concentration units were reported in the literature

Garlic component/preparation	Organism	MIC (mg/L)	Reference
Allicin	*H. pylori*	6–12	[23]
	*Pseudomonas* spp.	64	[10]
	*Streptococcus* spp.	64	
	*Staphylococcus* spp.	64	
	*P. aeruginosa*	64	
	*S. pneumonia*	32	
	*S. pyogenes*	32	
	*S. aureus*	32	
	*A. baumannii*	16	
	*E. coli*	23	[11]
DAS1	*H. pylori*	2074–4148	[23]
	*B. cereus*	64	[24]
	*C. jejuni*	56	
	*C. botulinium*	64	
	*E. coli*	72	
	*L. monocytogenes*	48	
	*S. enteric*	54	
	*S. aureus*	64	
	*V. cholerae*	72	
DAS2	*S. aureus*	2	[23]
	*H. pylori*	100	
	*B. cereus*	14	[24]
	*C. jejuni*	12	
	*C. botulinium*	20	
	*E. coli*	20	
	*L. monocytogenes*	8	
	*S. enteric*	12	
	*S. aureus*	16	
	*V. cholerae*	24	
DAS3	*S. aureus*	0.5	[23]
	*H. pylori*	13–25	
	*B. cereus*	4	[24]
	*C. jejuni*	2	
	*C. botulinium*	4	
	*E. coli*	12	
	*L. monocytogenes*	2	
	*S. enteric*	2	
	*S. aureus*	8	
	*V. cholera*	12	
	*M. tuberculosis*	2.5	[25]
DAS4	*H. pylori*	3–6	[23]
	*B. cereus*	1	[24]
	*C. jejuni*	1	
	*C. botulinium*	1	
	*E. coli*	4	
	*L. monocytogenes*	0.5	
	*S. enteric*	0.5	
	*S. aureus*	2	
	*V. cholerae*	4	
Garlic oil^a^	*H. pylori*	8–32	[23]
	*B. cereus*	40	[24]
	*C. jejuni*	36	
	*C. botulinium*	32	
	*E. coli*	48	
	*L. monocytogenes*	20	
	*S. enteric*	32	
	*S. aureus*	36	
	*V. cholera*	40	
	*S. boydii*	2.75	[26]
	*S. flexnar*	2.75	
	*S. fluvialis*	2.75–5.5	
	*V. metschnikovii*	0.34	
	*V. parahaemoyticus*	0.08	
	*V. enterocolittica*	0.68	
	*C. coli*	0.49	
	*C. lari*	0.49	
	*B. fragilis*	0.04	
	*B. subtilis*	0.17–0.68	
	*E. aerogenes*	0.68	
	*E. faecalis*	0.34	
	*K. aerogenes*	0.17	
	*P. vulgaris*	2.74	
	*L. acidophilus*	0.34–2.75	
	*S. faecalis*	0.34	
	*S. mutans*	0.08	
	*S. pyogenes*	0.04	
Garlic extract^a^	*P. gingivalis*	16.6	[27]
	*P. aeruginosa*	6	
	*A. actinomycetemcomitans*	62.5	
	*S. aureus*	4	[28]
	*E.coli*	7	
	*B. subtilis*	0.1	[29]
	*K. pneumonia*	0.2	
	*S. epidermidis*	0.9	
	*S. typhi*	0.02	
	*Proteus* spp.	7–21	[30]
	*H. pylori*	2–5	[31]
	*S. epidermidis*	22.9	[32]
	*S. pneumoniae*	30.3	
	*S. pyogenes*	33	
	*H. influenzae*	30.5	
	*Shigella* spp.	15.6	
	*P. aeruginosa*	3.5	
	*S. mutans*	4–32	[33]

^a^Activity depends on how garlic oil and garlic extract are manufactured. Various papers depicting the biological activity of polysulfanes did not mention the characterization of such preparations. The concentrations of individual polysulfanes in an extract or oil widely depend on method of extraction or distillation

**Table 2:** MIC (mg/L) of polysulfanes against different pathogenic fungal species. MIC values have been converted to mg/L where other concentration units were reported in the literature

Garlic component/preparation	Organism	MIC (mg/L)	Percentage inhibition at MIC (%)	Reference
Allicin	*C. albicans*	0.8	100	[34]
	*C. neoformans*	0.3		
	*C. parapsilosis*	0.15		
	*C. tropicalis*	0.3		
	*C. krusei*	0.3		
	*T. glabrata*	0.3		
	*Aspergillus* spp.	8–32	100	[35]
	*T. rubrum*	12.5	90	[36]
DAS2	*C. krusei*	8	100	[14]
	*C. albicans*	1		
	*C. krusei*	8		
	*C. glabrate*	4		
	*A. niger*	2		
	*A. fumigates*	8		
	*A. flavus*	4		
DAS3	*C. krusei*	2	100	[14]
	*C. albicans*	0.5		
	*C. krusei*	4		
	*C. glabrate*	2		
	*A. niger*	1		
	*A. fumigates*	4		
	*A. flavus*	2		
Garlic oil^a^	*C. albicans*	0.35	100	[20]
	*P. funiculosum*	0.69		
Garlic extract^a^	*Candida* sp.	14.9	100	[32]

^a^Activity depends on how garlic oil and garlic extract are manufactured. Various papers depicting the biological activity of polysulfanes did not mention the characterization of such preparations. The concentrations of polysulfanes in an extract or oil widely depend on method of extraction or distillation

Garlic was tested for synergistic effects with antibiotics (levofloxacin, gentamicin, azithromycin, and doxycycline) against *Pseudomonas* and *Acinetobacter* genera. This results in a decrease in the antibiotic MIC of 4-≥32, 4-≥2048, 2-≥2048 and 2-≥128 fold, respectively. The garlic increased the rate of lethality of the antibiotics against the bacteria. While these results show a potential for the synergistic use of garlic with antibiotics, a notable weakness of this study is it does not provide any details of the garlic preparation that is used [37, 38]. This unfortunately limits the meaningfulness of this piece of work.

#### Anticancer Activities

Polysulfanes have also been studied as a potential anticancer agent. Most studies have been conducted on DAS3 which shows this molecule as promising chemopreventive therapy for cancer. Studies with polysulfanes on different cancer lines and effective dose rates are summarized in Table 3. This research area has developed in the last decade enormously and various research groups have identified different targets in different types of cancer cell lines. The details of molecular targets identified by polysulfanes are highlighted in next section. This was noticed that some cancer cell lines are more sensitive to polysulfanes than others (Table 3). For example, DAS3 was effective at very low concentration (~ 2–9 mg/mL) in colon and breast cancer compared to gastric and skin (~ 29 mg/mL).

**Table 3:** Effect of polysulfanes on different human cancer cell lines and their molecular targets. The concentration units reported in the literature have all been converted to mg/mL so they can be compared with the antimicrobial activities in Tables 1 and 2

Redox modulator	Cancer type/cell line	Dose (mg/mL)	Target	Effect	Reference
Ajoene	Leukemia	9.36	Bcl-2	Inhibition of proliferation and induction of apoptosis	[39]
	HL-60, U937, HEL and OCIM-1		Caspase-3		
DAS2	Breast cancer	2.6	Estrogen receptor (ER)-positive (KPL-1 and MCF-7) and -negative (MDA-MB-231 and MKL-F)	Growth inhibition of cancer cells by inducing apoptosis	[40]
	MDA-MB-231				
DAS2	Breast	29.2	Kinase protein	Inhibited proliferation of MCF-7 cells and increased apoptotic ratio	[41]
	MCF-7				
			Caspase-3		
DAS2	Colon	29.2	Histone H3 and H4	Inhibition of caner proliferation by interaction with HDAC pathway	[42]
	Caco-2, HT-29				
DAS3	Liver	35	Caspase-3	Increased H_2_O_2_ levels, lowered thiol levels and inhibited cell proliferation	[3]
	HepG2				
DAS3	Colon	2.0	Tubulin	Suppression of cell growth	[43]
	HCT-15	2.3			
	DLD-1				
DAS3	Prostate	7.1	CDK1	Inhibition of cells by dose dependent manner.	[44]
	PC-3				
DAS3	Gastric	20.5	Bcl-2	Inhibited viability of BCG-823 in vitro and modulated Bcl-2.	[45]
	BGC-823				
DAS3	Breast	1.78	MMP2/9	Suppressed metastasis	[46]
	MDA-MB-231				
	HS 578t				
DAS3	Colon	8.91	Focal adhesion kinase (FAK	Inhibition of angiogenesis	[47]
	HT29				
DAS3	A375	17.8	Mitochondrial caspase pathway	Increase in ROS	[48]
	Skin				
DAS3	Lymphoma	3.56	NF-κB	Apoptosis in primary effusion lymphoma [PEL]	[49]
	BC2, BC3,				
	BCBL1, HBL6				
DAS3	Prostate	3.56	Androgen receptor (AR)	Decrease in AR levels	[50]
	LNCaP, C4-2, TRAMP-C1				
DAS3	Glioblastoma	17.8	Bcl-2	Inhibition and proliferation	[51]
	U87MG				
	Neuroblastoma				
	SH-SY5Y				

General reviews have been published on polysulfanes as chemopreventives with a special focus on DAS3 for pediatric cancer treatment. A recent review indicated that DAS3 is of importance not only for its remarkable antitumor and cancer preventive effects, as suggested by many in vitro and in vivo studies, but also because of its many health benefits, like improvements to immune-system function, radioprotection, and protection against microbial infections. These features make it a potential candidate for the treatment of pediatric cancers. Herman-Antosiewicz and Yi concluded the molecular targets of polysulfanes in cellular environment showing that it selectively targets the cancer cells [52, 53].

DAS3 is more potent than mono- and disulfides against skin cancer. DAS3 inhibits cell growth of human melanoma A375 cells and basal cell carcinoma (BCC) cells by increasing the levels of intracellular reactive oxygen species (ROS) and DNA damage and by inducing G2/M arrest, endoplasmic reticulum (ER) stress. A recent review focuses on the molecular mechanisms of garlic-derived allyl sulfides on skin cancer prevention [54]. Similar findings were observed in human colon cancer cells HCT-15 and DLD-1. The growth of the cells was significantly suppressed by DAS3, but neither DAS nor DAS2 showed such an effect [55]. An interesting paper depicts that allyl group in polysulfanes are responsible for the disruption of microtubule network formation in human colon cancer cell line HT-29 cells [43]. The effective dose rate in different cancer cell lines and their molecular targets are summarized in Table 3 below.

### Molecular Targets and Metabolism of Sulfanes

Low molecular weight (LMW) thiols such as glutathione (GSH) serve as intracellular redox buffers in most aerobic organisms. They play a central role in the essential maintenance of an intracellular reducing environment and neutralize the damaging effects of toxic oxidants. When the cellular concentrations of these LMW thiols are dramatically reduced, the ability to defend against, and survive, oxidative stress is severely impaired [56].

Upon entering the target (i.e., microbes and cancer cells) polysulfanes undergo rapid thiol-polysulfide exchange reactions with these LMW thiols. The implications of this process are twofold: (a) cellular LMW thiol concentrations decrease making them more susceptible to oxidative stresses and (b) in parallel, RSS such as allyl persulfide, hydropolysulfide species are formed, which can enhance the production of toxic oxidants (e.g., hydrogen peroxide, superoxide) thereby increasing oxidative stress [2].

**Fig. 2:**
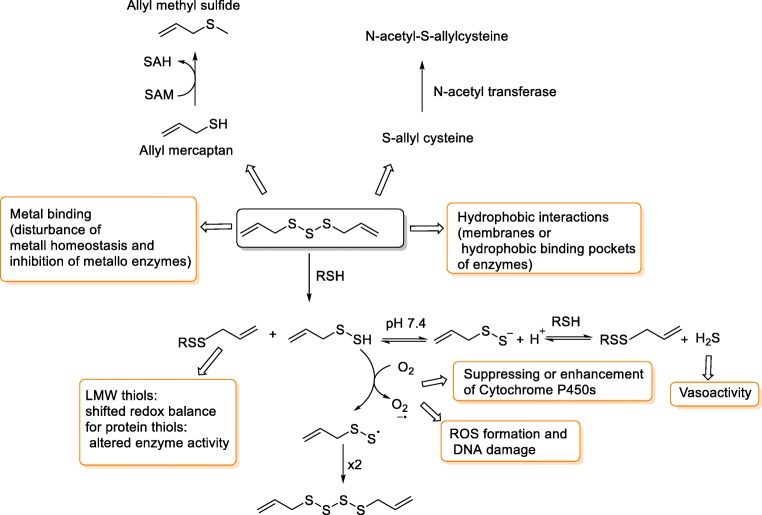
A summary of diallyl polysulfane reactions/interactions in vivo and their physiological consequences [1]. Once polysulfanes enter the target cell, they can react with thiols (GSH) to produce hydropolysulfane (RSxH), which upon oxidation (ROS) can produce perthiyl radicals (RSS•). Perthiyl radicals can decompose into thiyl radicals (RS•), after accepting an electron they can result in formation of thiols. The pathways of such species have been discussed previously, and hence, will not be presented here [2, 4]. Diallyl polysulfanes can either enhance or suppress cytochrome P450s, which are involved in the detoxification process [59]. It is hypothesized that reduction of polysulfanes leads to the production of allyl mercaptan (AM) which can be further methylated by *S*-adenosyl methionine synthetase into allyl methyl sulfide (AMS) [60, 61]. AM and AMS have been determined as DAS1 and DAS2 metabolites [62]. SAM *S*-adenosyl methionine, SAH *S*-adenosyl homocysteine

In addition, polysulfanes can also react with exposed cysteine thiols on proteins to form *S*-allyl modified proteins. Such cysteinyl-*S*-allylations processes can result in altered/impaired protein function [57]. In addition to the redox activity of these molecules, their lipophilicity may contribute to their biological activity (e.g., by interrupting membrane integrity, binding to hydrophobic sites on proteins) [18, 58]. These different modes of action are summarized in Fig. 2.

In pathological conditions, it has been shown that organosulfur compounds from garlic have protective effects, which are mostly associated with antioxidant properties. Because of the ability of polysulfanes to target multiple biochemical pathways, many researchers have studied their roles. The major metabolic pathways of sulfanes in mammals include methylation, oxidation, glutathione, and *N*-acetyl conjugations, and in certain instances, after ingestion of raw garlic by a human, allyl methyl sulfide, allyl methyl disulfide, DAS2, and DAS3 were discovered in the breath of tested volunteers [63], therefore providing an early example of their biochemical transformations within the body.

Recent studies in cancer cell lines investigated various pathways of polysulfanes within cell that includes proliferation, G2/M phase arrest, and radical and triggering antioxidant response [41, 52, 64–66]. Polysulfanes can also activate other biochemical pathways, i.e., inducing caspase-3 activity, enhancing H_2_O_2_ levels, and strongly decreasing glutathione levels, inhibiting the expression of estrogen proteins [67–71], modulation of Bcl-2 family proteins [48], inhibition of HDAC pathways [72], and angiogenesis [47].

The metabolic and fate of polysulfanes have not been widely studied, but this subject has gathered attention in the last few years. In relation to DAS1, it can form conjugates with GSH and modified glutathione *S*-transferase, glutathione peroxidase, and glutathione reductase activities [73]. Some studies in rats conclude that DAS2 quickly produces allyl mercaptan, allyl methyl sulfide, allyl methyl sulfoxide, and allyl methyl sulfone as major metabolites in rat liver. Similarly, allyl mercaptan was isolated from rat liver and extracellular fluid of primary rat hepatocytes when perfused with DAS2 (1 mM) after 90 min [74].

DAS2 and DAS3 can induce NAD(P)H:quinone oxidoreductases 1 (NQO1) via nuclear factor erythroid 2 (Nrf2) activation [75–78]. It has also been shown that DAS3 can induce intracellular ROS accumulation, which would activate Nrf2 by oxidation of cysteine residues [77]. DAS3 can also elevate intracellular ROS levels and the redox-regulatory proteins, such as glutaredoxin (GRX) [79]. In human cancer cells, this activates the ASK1-MEK-JNK-Bim transduction-signaling pathway, which subsequently triggers the Bax-dependent mitochondrial apoptotic pathway resulting in apoptosis [80, 81].

As with all polysulfanes, increasing the H_2_O_2_ concentration can result in a cascade of molecular events due to specific oxidation of signaling proteins including kinases, transcription factors, and phosphatases. H_2_O_2_ can also react with low molecular weight thiols, such as GSH and cysteine, by a nucleophilic attack from the thiolate onto the reactive H_2_O_2_. The acidity of the electrostatic environment around the –SH group may increase which increases the reactivity towards H_2_O_2_. This would result in a higher fraction of the thiolate form. In cysteine residues, lower stability of the thiolate increases nucleophilicity towards H_2_O_2_ of the thiolate [82]. Some studies using a sulfane model suggest an important role for O_2_•- radicals in inducing cell death (apoptosis) [83].

Polysulfanes have also been investigated as potent H_2_S donors in the presence of thiols [84, 85]. Preclinical studies have shown that enhancement of endogenous H_2_S has an impact on vascular reactivity. In CVD models, the administration of H_2_S prevents myocardial injury and dysfunction. It is hypothesized that these beneficial effects of garlic may be mediated by H_2_S-dependent mechanisms [86]. Computational and experimental studies have revealed that glutathione and cysteine are capable of releasing H_2_S from diallyl trisulfide (DAS3), via thiol-polysulfide exchange pathways, but diallyl disulfide (DAS2) is a much poorer H_2_S donor via an α-carbon nucleophilic substitution pathway [87, 88].

Intraperitoneal (*ip*) administration of DAS1, DAS2, and DAS3 in mice increased the activity of rhodanese. Moreover, DAS2 and DAS3 increased the total sulfane sulfur level and γ-cystathionase activity in the normal mouse kidney. Aldehyde dehydrogenase activity was inhibited in the kidney after DAS3 administration. The results indicated that none of the studied polysulfanes affected the level of bound sulfur or H_2_S. Thus, it can be concluded that garlic-derived DAS2 and DAS3 can be a source of sulfane sulfur for renal cells but they are not connected with persulfide formation [89]. DAS1, DAS2, and DAS3 dissolved in corn oil were given intraperitoneally to mice for 10 days. It showed that polysulfanes had a beneficial effect in the mouse liver, decreasing reactive oxygen species and malondialdehyde levels, and increasing glutathione S-transferase activity and non-protein sulfhydryl group level. Moreover, DAS2 and DAS3 elevated the total sulfane sulfur pool and activity suggesting its antioxidant and regulatory capacities [90].

Some studies of DAS4 have shown that it induces reactive oxygen species (ROS) in normal cells similar to cancer cells in a time (0 to 60 min) and dose-dependent manner (0 to 50 μM) [91]; it also activates both the eIF2α and Nrf2/HO-1 pathways [92].

## Conclusion

This review summarizes the numerous biological activities garlic diallyl polysulfanes are involved in. While there is extensive evidence that show the various medicinal benefits of garlic polysulfanes, there is a critical need for controlled clinical studies to strictly evaluate the safety and efficacy of these compounds for establishing sufficient application methods before medical use. Furthermore, polysulfanes are emerging as promising, environmentally benign pesticides which are not only safe for humans, but also various non-target species endangered by synthetic chemical pesticides [8, 93]. With further work, diallyl polysulfanes could provide the basis for the innovative development of novel antibiotics, fungicides, pesticides, and anticancer agents.
